# *In vitro* delivery and transwell uptake of volatile and semi-volatile organic compounds in an air–liquid interface exposure system

**DOI:** 10.3389/ftox.2026.1783871

**Published:** 2026-03-26

**Authors:** Ji Hyun Lee, Hongku Lee, Kang Ho Ahn, Mi Seong Jo, Jin Kwon Kim, Youngshang Han, Yong Taek Kwon, Elaine M. Faustman, Il Je Yu

**Affiliations:** 1 HCT Seattle, Seattle, WA, United States; 2 HCTm, Icheon, Republic of Korea; 3 Department of Mechanical Engineering, Hanyang University, Ansan, Republic of Korea; 4 H&H Bio, Asan, Republic of Korea; 5 Department of Mechanical Engineering, University of Washington, Seattle, WA, United States; 6 Department of Environmental and Occupational Health, University of Washington, Seattle, WA, United States

**Keywords:** air-liquid interface, delivery, diffusion, *in vitro* inhalation, organic solvents, VOCs

## Abstract

**Introduction:**

Volatile organic compounds (VOCs) readily penetrate the respiratory tract and cross the air–liquid interface (ALI), yet their in vitro deposition behavior and dose uniformity remain poorly characterized.

**Methods:**

In this study, we evaluated the delivered concentration in the basolateral compartment and inter-transwell variability of isopropyl alcohol (IPA), acetone, and benzyl alcohol (BA) in a six-transwell ALI exposure system. VOCs were generated by atomization at airflow rates of 0.2−3 L/min and delivered for 1–2 h. Delivered concentrations were quantified using photoionization detection or UV–Vis spectroscopy, and aerosol characteristics were monitored by scanning mobility particle sizing.

**Results:**

Despite rapid evaporation and predominantly vapor-phase transport of IPA and acetone, and semi-volatile aerosol BA, VOC delivery to the ALI was reproducible and spatially uniform. IPA and acetone exposures produced coefficients of variation (CVs) below 10% and 16%, respectively, across transwells, whereas benzyl alcohol exhibited higher variability (CV ≤ 19%) due to its lower vapor pressure and higher viscosity. No statistically significant positional differences were observed under any exposure condition.

**Discussion:**

These findings indicate that volatile organic compounds delivered to the ALI undergo diffusion-driven gas–liquid delivery, resulting in a relatively uniform spatial distribution. This study provides exposure-relevant delivery characterization for VOCs and semi-VOCs in ALI systems, supporting future NAM-based inhalation studies without implying biological dose equivalence.

## 1 Introduction

VOCs are widely used in industrial, laboratory, and consumer products, and inhalation is a major route of human exposure. Upon entering the respiratory tract, volatile and semi-volatile organic compounds can cross the alveolar–capillary barrier and distribute systemically, leading to both acute and chronic health effects, including central nervous system depression, hepatic dysfunction, and respiratory irritation. The dynamics of solvent uptake in the lung depend on the physicochemical properties of the solvent, particularly vapor pressure, solubility, and diffusivity, which govern partitioning across the air–liquid interface (ALI) (World Health Organization, 2000; Agency for Toxic Substances and Disease Registry, 1997).

Inhalation toxicity testing is costly and resource-intensive, driving the adoption of new approach methodologies (NAMs), including *in vitro* and *in silico* test systems, to improve efficiency and ethical standards (Kleinstreuer et al., 2016; EPA, 2021). *In vitro* ALI exposure systems provide a controlled platform for simulating inhalation exposure while reducing the use of animals in toxicology testing. These systems allow direct contact of airborne materials with cultured lung cells or liquid interfaces, enabling precise quantification of deposited or delivered doses. To ensure relevance to *in vivo* outcomes, *in vitro* NAM-based test systems must be supported by dosimetry and dose metrics equivalent to those used *in vivo* tests (Lee et al., 2025a). Among these, air–liquid interface (ALI) systems expose the apical cell surface to aerosols (the term aerosol includes both the particles and the suspending gas, which is usually air; particle size ranges from about 0.002 to more than 100 ㎛, Hinds, 1999), usually under controlled airflow while maintaining contact with the basal medium. ALI platforms include flow-through systems (e.g., Vitrocell®) and alternatives based on electrostatic precipitation, gravitational settling, or hybrid deposition mechanisms (Lucci et al., 2018; Jeannet et al., 2015; Savi et al., 2008; de Bruijne et al., 2009; Lenz et al., 2009; Aufderheide and Scheffler, 2013). While air–liquid interface (ALI) exposures using transwell systems have become a standard method for simulating inhalation of particulate aerosols and nanomaterials *in vitro* (Lenz et al., 2009; Aufderheide and Mohr, 2004; de Bruijne et al., 2009; Lenz et al., 2014; Mulhopt et al., 2016), most studies have primarily focused on establishing physiologically relevant exposure conditions and quantifying delivered dose. However, comparatively little attention has been paid to evaluating inter-transwell deposition variability or deposition homogeneity, parameters essential for ensuring the reproducibility and reliability of *in vitro* inhalation toxicology data. Although recent work has begun to address spatial uniformity and measurement accuracy across exposure wells (Polk et al., 2019; Gkanis et al., 2021), the systematic assessment of deposition reproducibility between wells within a single exposure run remains limited, highlighting a methodological gap in the current ALI testing frameworks.

Several studies have examined organic solvents for VOCs. Organic solvent 1,3-dichloropropene (1,3-DCP) using an *in vitro* and *in silico* approach to assess inhalation toxicity. Using human airway epithelial models representing five respiratory regions, researchers measured cytotoxicity and barrier integrity after exposure and applied dosimetry modeling to predict *in vivo* outcomes. Predicted toxicity thresholds aligned well with animal data, showing a gradient of sensitivity from nasal to alveolar tissues. The results support these integrated methods as viable alternatives to traditional animal testing for inhalation safety assessment (Moreau et al., 2022). Benchmark Dose Modeling Approaches for Volatile Organic Chemicals (VOCs) applied using a novel *in vitro* air–liquid interface (ALI) exposure system for assessing the toxicity of volatile organic chemicals (VOCs) under physiologically relevant conditions. Using BEAS-2B and primary human bronchial epithelial (pHBEC) cells, researchers evaluated cytotoxicity, cell viability, and gene-expression changes following exposure to several VOCs. Benchmark dose modeling of gene expression identified sensitive molecular responses that aligned closely with known human and animal toxicity data. The findings support this ALI-based approach as a promising method for determining molecular points of departure in airway toxicity testing of volatile chemicals (Speen et al., 2022). Jackson et al. (2025) compared transcriptomic responses to volatile organic chemicals (VOCs) between *in vitro* human airway models and *in vivo* mouse inhalation exposures. Using whole-transcriptome and physiologically based toxicokinetic (PBTK) modeling, researchers found largely comparable (<2-fold) points of departure across systems, except for dichloromethane, which differed due to species-specific metabolism. Despite distinct gene expression profiles, Hmox1 was consistently upregulated across models. These findings demonstrate the value of NAM-based test systems when coupled with refined internal dose modeling for improving in vitro–in vivo extrapolation (IVIVE) in inhalation toxicity assessment. The workshop, organized by the American Cleaning Institute (ACI), reviewed the use of *in vitro* new approach methodologies (NAMs) to assess respiratory irritation, highlighting the importance of exposure characterization and dosimetry to support NAM-derived data in human health risk assessment. Experts discussed exposure systems, cell and tissue models, and integration of NAM data with existing toxicological information. Key outcomes included the need for standardized assay characterization, improved dosimetry and exposure relevance, and guidance to align NAM-derived points of departure (PODs) with current risk assessment practices. The proceedings highlight priorities for advancing and harmonizing *in vitro* methods in respiratory safety evaluation (Haber et al., 2024).

While ALI exposure has been extensively applied to particulate aerosols and nanomaterials, the *in vitro* delivery characteristics of volatile organic chemicals with high evaporation rates and rapid diffusion have been seldom studied. Isopropyl alcohol (IPA) and acetone are representative water-miscible solvents with relatively high vapor pressures and rapid evaporation rates, and benzyl alcohol is also a water-soluble solvent with low vapor pressure and high viscosity. Understanding their delivery behavior may provide essential information for interpreting *in vitro* exposure results and correlating them to real-world inhalation scenarios.

In this study, we conducted a series of *in vitro* VOC aerosol exposure studies to characterize delivery and inter-transwell delivery to the basolateral compartment, using IPA, acetone, and BA aerosols on 6-transwell ALI plates under different generation flow rates and exposure durations. We quantified delivered VOC concentrations and assessed the homogeneity of delivery (or inter-transwell variation) across transwells. The goal was to determine whether volatile solvent aerosols can achieve consistent, measurable delivery to the ALI, thereby providing foundational data for future studies of solvent inhalation toxicity. Although air–liquid interface exposure systems are frequently discussed within the context of NAM-based inhalation testing, the present study does not introduce a new toxicological test method; rather, it focuses on characterizing delivery and deposition variability to support interpretation of *in vitro* inhalation studies.”

## 2 Materials and methods

### 2.1 Generation of test aerosol

**FIGURE 1:**
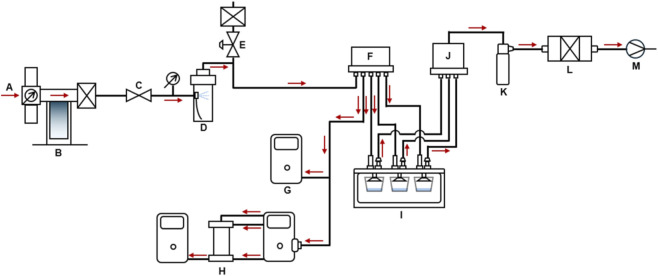
VOC aerosol generation scheme. Arrows indicate air flow direction. **(A)** fresh air; **(B)** air cleaner; **(C)** valve; **(D)** atomizer; **(E)** valve; **(F)** diffuser; **(G)** PID; **(H)** SMPS, **(I)** HIVIS; **(J)** 6-way collector; **(K)** impinger; **(L)** sampling cassette; **(M)** pump.

Isopropyl alcohol (IPA, ForPro, item #140258), acetone (ForPro, item #140024), and benzyl alcohol (BA, DMSO Store Inc.) were aerosolized using purified air as the carrier gas. Their chemical properties are shown in Table 1. A mass flow controller (MFC, AERA, FC-7810CD4, V, Tokyo, Japan) was used to generate organic solvent aerosol with air flows of 0.2-3 LPM (L/min). Figure 1 shows the schematic of the solvent aerosol generation and exposure system.

**TABLE 1:** Chemical properties at 20 °C.

VOCs	Water solubility	Vapor pressure	Viscosity	Reference
Isopropyl alcohol	Miscible in all proportions with water	∼44 mmHg (≈5.9 kPa	∼2.4 mPa·s	www.intersurfchem.com
Acetone	Miscible in all proportions with water	∼181.7 mmHg (≈24.2 kPa)	∼0.32 mPa·s	https://macro.lsu.edu/HowTo/solvents/acetone.htm?utm_source=chatgpt.com
Benzyl alcohol	∼4.29 g/100 mL	∼0.167 mmHg (≈0.022 kPa)	∼5.5–5.8 mPa·s	Registration https://echa.europa.eu/registration-dossier/-/registered-dossier/14748/4/9Dossier - ECHA

**FIGURE 2:**
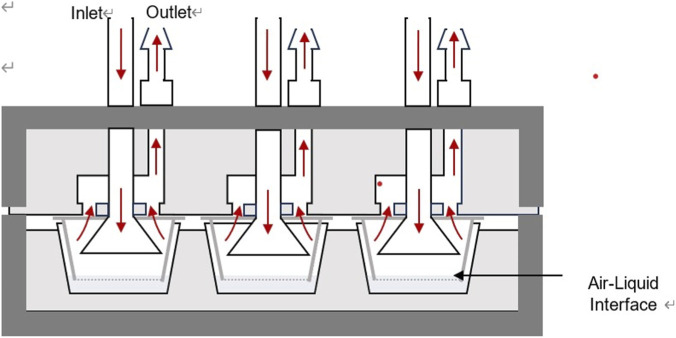
Exposure and exhaust funnel to transwells in HIVIS. Arrows indicate flow direction.

The generated VOC aerosols were diluted in a diffuser with five nozzles and delivered to a HIVIS (HCT *In Vitro* Inhalation System, Figure 2), which delivered them into a 6-transwell. The exhausted air was passed through an impinger to measure the air concentration. The flow rate to the exhausted sampling cassette was 30 mL/min, assuming 5 mL/min per transwell, respectively, using a low flow sampling pump (Gilian LFS-113, Sensidyne, St. Petersburg, FL), which was previously calibrated by a Bios calibrator (Dry Cal DC Lite, Butler, NJ). The HIVIS system accommodated a commercial 6-transwell plate (Falcon cat. 353046) with inserts (0.4 ㎛ transparent Polyethylene terephthalate (PET) membrane, cat. 353090), and each transwell was fitted with an axial-flow inlet funnel and an outlet for exhausted air (Figure 2). A 6-well plate containing cell culture inserts, with 2 mL of water below the inserts, was exposed to VOCs for various durations. We used 100% isopropyl alcohol and acetone to generate VOC aerosols, and 30% benzyl alcohol in ethyl alcohol to reduce viscosity and improve aerosol generation. We used the acellular system to monitor the delivery of VOCs to the ALI. The exposure period was initially 2 h for VOCs. Still, it changed to 1 h for acetone due to rapid consumption of the test material, and benzyl alcohol due to the relatively high viscosity. High-viscosity aerosols tend to form larger, less stable droplets, leading to increased wall losses and reduced delivery efficiency, resulting in lower and more variable delivered doses compared with low-viscosity aerosols (Gou et al., 2025). Therefore, the diffuser condensates benzyl alcohol aerosols, and all tubes from the atomizer to the HIVIS and the collector were cleaned with water and dried after exposure to prepare for the next exposure experiment. All test material generation and monitoring are conducted in a fume hood.

### 2.2 Droplet evaporation rate estimation

The droplet evaporation rate was calculated based on the formula

$$d^{2}\left(t\right)=d_{0}^{2}-\text{Kt}$$

where:

- d_0_ = initial diameter
- K = evaporation constant (m^2^/S; depends on vapor pressure, diffusivity, temperature)
- d(t) = droplet constant at time t
- t = time

### 2.3 Monitoring of *in vitro* inhalation and analysis of aerosols

The distribution and maintenance of VOC aerosols in terms of size and number were measured directly using an SMPS (size range: 9–294 nm; ART Plus, Icheon, Korea), which monitored particle diameter and number during the exposure periods. The air samples were collected into 20 mL of water in an impinger at the HIVIS outlet port using a Gilian flow sampler pump at a flow rate of 30 mL/min.

### 2.4 Determination of delivered concentration to the basolateral compartment

This study was not designed to achieve absolute mass closure relative to the mass initially nebulized. Unavoidable losses due to evaporation, adsorption onto tubing, and interactions with the chamber wall are inherent to exposure systems involving volatile organic compounds. Accordingly, this work focuses on relative delivery efficiency and inter-transwell variability under controlled exposure conditions, rather than on complete mass balance.

An impinger-based analytical method was employed to enable rapid and quantitative determination of volatile organic compounds (VOCs) transferred to the basolateral compartment. This approach was selected because highly volatile compounds cannot be reliably quantified using conventional gravimetric or filter-based methods. The impinger method allows real-time detection of VOCs aerosolized from aqueous media using a photoionization detector (PID), thereby minimizing analytical losses associated with solvent extraction or chromatographic separation.

**FIGURE 3:**
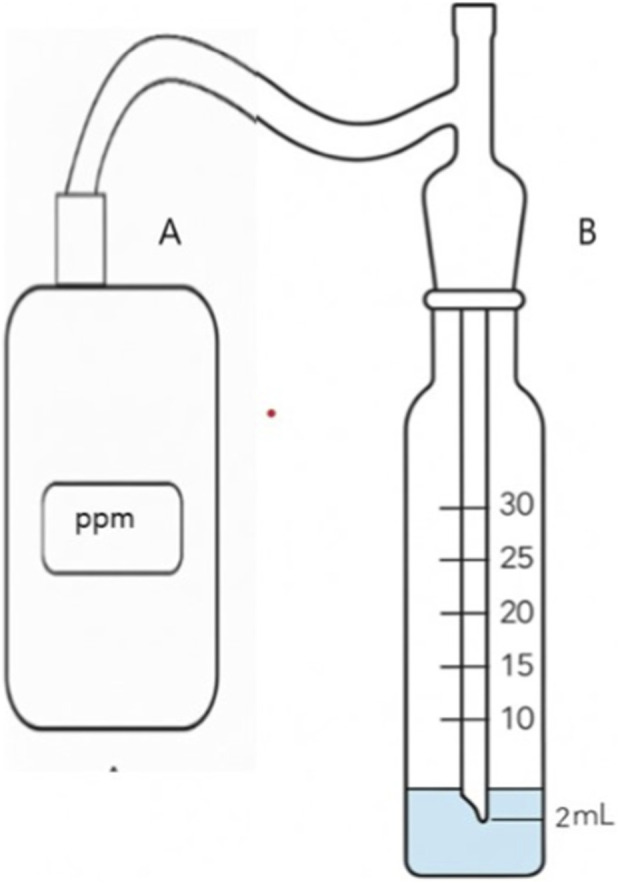
VOC analysis using an impinger and a PID. **(A)** PID; **(B)** Impinger. Air drawn from the impinger containing 2 mL of sample was analyzed by PID. The entire 2 mL of basolateral medium was collected and transferred to an impinger **(B)**. The liquid was aerosolized using a sampling pump **(A)**, and the resulting VOC-containing aerosol was analyzed in real time by the PID. VOC concentrations in the basolateral compartment were determined using the corresponding calibration curves.

**FIGURE 4:**
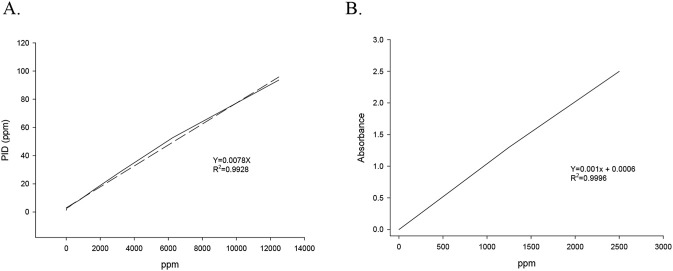
Standard curves to determine volatile organic chemical deposition to the transwell. Solid line, measured; dotted, regression. **(A)** Isopropyl alcohol and acetone. **(B)** Benzyl alcohol.

To generate calibration curves, serial dilutions of each VOC were prepared, and 2 mL of each diluted solution was added to an impinger. Air exiting the impinger was sampled into a PID (Industrial Scientific iBRID MX6; flow rate 0.3 L·min^-1^), and PID responses were recorded to construct standard curves (Figures 3, 4A). For exposure experiments, transwells containing 2 mL of water in the basolateral compartment were exposed for 1–2 h. Following exposure, the entire 2 mL of basolateral medium was collected and transferred to an impinger. The liquid was aerosolized using a sampling pump (Figure 3), and the resulting VOC-containing aerosol was analyzed in real time by the PID. VOC concentrations in the basolateral compartment were determined using the corresponding calibration curves.

Isopropyl alcohol and acetone concentrations were quantified using PID-based standard curves (Figure 4A), constructed by plotting known solution concentrations against PID detector responses (ppm). Benzyl alcohol concentrations were quantified using a UV–Vis spectrophotometer at 265 nm, corresponding to the maximum absorbance of the benzene ring; the calibration curve is shown in Figure 4B.

Percent delivery was calculated as the relative metric (mean VOC concentration in the basolateral compartment divided by the corresponding air concentration at the impinger) × 100. This metric is intended to assess relative delivery efficiency and inter-transwell variability and does not represent a biological dose or absolute deposited mass. Air concentrations were derived from impinger-captured VOC concentrations, accounting for the total sampled air volume and applying appropriate unit conversions. All experiments were conducted under controlled environmental conditions at 20 °C and 40%–60% relative humidity.

### 2.5 Statistical analysis

The statistical analysis was performed using SPSS version 22.0 (IBM, New York, United States). Data were presented as mean ± standard deviation (SD) and the coefficient of variation (CV).

The statistical analysis was performed using ANOVA for the 6-transwell data from Table 3 to Table 5.

## 3 Results

### 3.1 Aerosol number. size distribution and maintenance of concentration during the exposure period

**FIGURE 5:**
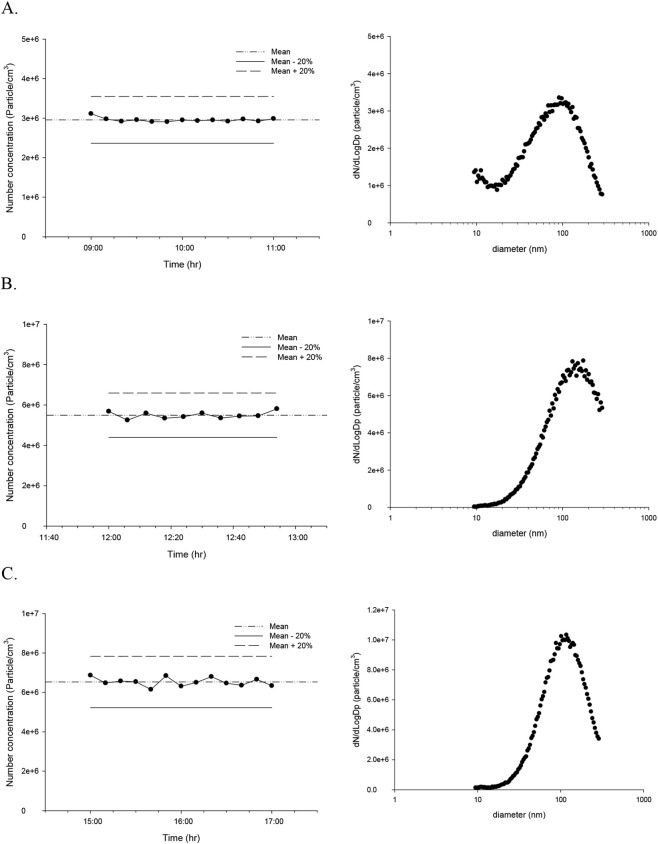
Maintenance of aerosol concentration during the exposure period and aerosol size distribution. **(A)** Isopropyl alcohol. **(B)** Acetone. **(C)** Benzyl alcohol.

Although particle number and size distributions were monitored, most of the test compounds were present in the vapor phase and were not consistently detected by the SMPS. To estimate the air concentration in the transwell containing HIVIS, an impinger was used to collect VOCs and quantify their concentrations. Figure 5 shows IPA aerosol number and size distribution during a 2-h exposure period. The median diameter, geometric diameter, and geometric standard deviation (GSD) were 65.39 nm, 58.39 nm, and 2.45, respectively. Aerosol number concentration was 2.96 x 10^6^/cc. The median diameter, geometric diameter, and geometric standard deviation of acetone aerosol during 1-h exposure were 128.63 nm, 58.39 nm, and 1.78, respectively. Aerosol number concentration was 5.49 x 10^6^/cc. The VOC concentrations derived from particle number and size measurements by SMPS are shown in Table 2. Based on VOC concentrations calculated from particle number and size distributions, the majority of VOCs are likely in the vapor phase. The duration of the acetone exposure experiment was limited to 1 h due to the solvent’s rapid consumption. Benzyl alcohol aerosol median diameter, geometric mean diameter, and geometric standard deviation during a 2-h exposure period were 109.76 nm, 102.60 nm, and 1.92, respectively. The number concentration was 6.53 x 10^6^/cc.

**TABLE 2:** Particle size, number, concentration, and evaporation rate of VOCs.

VOCs	Density at 20 °C (g/mL)	Median (nm)	GM (nm)	GSD	Number (10^6^)	Mass concentration (mg/m^3^)	ppm	Evaporation time estimate (t)
Isopropyl alcohol	0.786	65.39	58.39	2.45	2.96	12.6	5	4–8×10^–6^ s
Acetone	0.79	128.63	121.64	1.78	5.49	21.6	8.95	8–16×10^–6^ s
Benzyl alcohol	1.045	109.76	102.60	1.92	6.54	32.04	7.13	0.24–1.2 s

GM, geometric mean; GSD, geometric standard deviation.

Evaporate rates of isopropyl alcohol and acetone aerosols are so fast that aerosols should be in the vapor phase when they are exposed to the basolateral compartment of the transwell (Table 2). The exposure period was initially 2 h, then changed to 1 h due to the relatively high viscosity of benzyl alcohol. High-viscosity aerosols tend to form larger, less stable droplets, leading to increased wall losses and reduced delivery efficiency, resulting in lower and more variable delivered doses compared with low-viscosity aerosols (Gou et al., 2025). Therefore, all tubes and diffuser were cleaned with water and dried after exposure to prepare for the next exposure experiment.

### 3.2 Delivery of IPA aerosols to 6-transwell

Table 3 presents the results of IPA delivery onto 6-well plates over a 2-h exposure period. Twelve delivery experiments were conducted at a flow rate of 30 mL/min. The average delivered concentration in each 6-well plate across 10 experiments was 13,491 ± 3712 ppm, with a coefficient of variation (CV) of 9.96% relative to the mean (Table 3). The average delivery rate to the basolateral compartment exceeded 100% of the total delivery rate. A one-way ANOVA showed no significant difference in delivered concentrations among the six trans-well positions, indicating uniform aerosol delivery across the exposure system. The delivered concentration estimate was 109.3 ± 28.2 of the exposed concentration (Table 3). It could be due to the saturation of IPA delivery to the basolateral compartment after 2 h exposure.

**TABLE 3:** Delivered IPA to the basolateral compartment (2 mL) of 6-transwell (MFC 3 LPM; 2-h exposure, 30 mL/min pump flow-rate) (n = 12).

Trans-well	1	2	3	4	5	6	Mean	SD	CV	Impinger conc.	Total mass in impinger	Total air volume sampled	Air conc	ppm in air	Delivery
Exp	ppm	ppm	ppm	ppm	ppm	ppm	ppm	​	%	ppm	mg	m^3^	mg/m^3^	ppm	%
1	10,811	11,495	11,298	9,022	9,324	9,232	10,197	1,127	10.96	3,601	72.02	0.0036	20,006	8,139	125.3
2	10,956	11,628	9,785	11,228	9,927	9,114	10,440	975	9.17	5,720	114.40	0.0036	31,778	12,928	80.8
3	7,996	10,220	11,746	10,956	12,785	11,128	10,805	1,623	15.02	3,996	79.92	0.0036	22,200	9,031	119.6
4	13,641	14,825	11,667	11,259	11,759	13,483	11,272	1,415	10.75	3,996	79.92	0.0036	22,200	9,031	124.8
5	10,897	11,936	12,256	11,179	12,295	11,077	11,607	628	5.41	3,846	76.92	0.0036	21,367	8,692	133.5
6	11,205	11,090	12,333	12,615	11,667	12,064	11,829	615	5.2	5,782	115.64	0.0036	32,122	13,068	90.5
7	14,038	10,987	11,243	12,949	14,541	14,730	13,098	1,664	13	5,833	116.66	0.0036	32,406	13,183	99.4
8	14,523	14,603	12,641	14,308	14,167	12,859	13,850	869	6.26	6,538	130.76	0.0036	36,322	14,777	93.7
9	19,122	15,570	14,581	13,500	17,000	13,108	15,513	2,281	14.7	5,270	105.40	0.0036	29,278	11,911	130.2
10	25,540	25,959	24,635	22,838	20,946	21,243	23,527	2,170	9.22	11,459	229.18	0.0036	63,661	25,899	90.8
11	11,429	14,280	12,614	13,600	14,200	14,643	13,461	1,223	9.09	9,914	198.28	0.0036	55,078	22,407	60.1
12	16,457	19,486	16,600	14,540	15,142	15,557	16,290	1755	10.77	4,429	88.58	0.0036	24,606	10,010	162.7
​	13,385 ±4,721	14,339 ±4,505	13,450 ±3,923	13,166 ±3,448	13,646 ±3,172	13,185 ±3,267	13,491 ±3,712	​	9.96 ±3.3	5,865 ±2,463	117 ±49	0.0036	32,585.2 ±13,687.8	13,256.4 ±5,568.5	109.3 ±28.2

SD, standard deviation; CV, coefficient of variation; % Delivery = (mean concentration of tanswell (ppm)/(ppm in air) x 100%.

### 3.3 Delivery of acetone aerosols to 6-transwell

Another VOC, acetone, was tested for its delivery on the ALI at a flow rate of 30 mL/min for 1 h. We selected a 1-h exposure because acetone was consumed at a higher rate than IPA. We tested at different MFC flow rates, from 3, 2, 0.5, to 0.2 LPM (Tables 4–7). The results show 7,196 ± 1808, 2,345 ± 293, 1,067 ± 188, and 2,277 ± 319 ppm at 3, 2, 0.5, and 0.2 LPM, respectively (Tables 4–7). Delivery of acetone to the basolateral compartment of transwell was 21.9 ± 6.5, 13.3 ± 1.9, 14.9 ± 3.2, and 7.7 ± 0.7 for 3, 2, 0.5, and 0.2 LPM, respectively, indicating the influence of generation rate. Furthermore, acetone delivery differed from IPA, likely due to differences in exposure duration and evaporation rate. The coefficient of variation (CV) of inter-transwell delivery was 11.73%, 13.78%, 15.8%, and 16.1% at 3, 2, 0.5, and 0.2 LPM, respectively, indicating a decreasing CV with increasing generation rate (Tables 4–7). A one-way ANOVA demonstrated no significant differences in delivered concentrations among the six trans-well positions, indicating uniform aerosol distribution across the exposure system.

**TABLE 4:** Delivered acetone to the basolateral compartment (2 mL) of 6-transwell (MFC 3 LPM; 1-h exposure, 30 mL/min pump flow-rate) (n = 9).

Trans-well	1	2	3	4	5	6	Mean	SD	CV	Impinger conc.	Total mass in impinger	Total air volume sampled	Air conc	ppm in air	Delivery
Exp	ppm	ppm	ppm	ppm	ppm	ppm	ppm	​	%	ppm	mg	m^3^	mg/m^3^	ppm	%
1	7,279	7,136	7,940	8,550	7,836	8,121	7,818	528	6.77	5,436	108.72	0.0018	60,400	25,427	30.7
2	7,879	7,836	7,279	7,963	7,279	7,836	7,634	282	3.69	6,135	122.70	0.0018	68,167	38,696	26.6
3	3,903	3,792	3,658	3,358	3,792	3,492	3,666	206	5.63	3,719	74.38	0.0018	41,322	17,395	21.1
4	6,548	6,548	5,781	5,992	6,103	5,003	5,996	575	9.59	6,214	124.28	0.0018	69,044	29,066	20.6
5	8,081	9,081	8,192	10,070	10,080	7,536	8,838	1,075	12.17	6,270	125.40	0.0018	69,667	29,328	30.1
6	7,421	7,564	6,150	7,135	8,135	5,864	7,045	872	12.38	6,271	125.42	0.0018	69,678	29,332	24.0
7	6,907	6,150	6,007	3,936	4,735	7,135	5,811	1,247	21.46	7,421	148.42	0.0018	82,456	34,711	16.7
8	8,835	10,107	10,678	6,435	11,378	8,864	9,566	1750	18.29	17,300	346.00	0.0018	192,222	80,920	11.8
9	7,007	8,979	9,964	8,835	8,978	6,579	8,390	1,308	15.60	11,378	227.56	0.0018	126,422	53,220	15.8
​	7,096 ± 1,382	7,466 ± 1878	7,294 ± 2,187	6,919 ± 2,238	7,591 ± 2,442	6,714 ± 2,442	7,196 ± 1808	​	11.73 ± 6.0	7,793.8 ± 4,114.1	155.9 ± 82	0.0018	86,598 ± 45,712	37,566 ± 19,027	21.9 ± 6.5

SD, standard deviation; CV, coefficient of variation; % Delivery = (mean concentration of tanswell (ppm)/(ppm in air) x 100%.

**TABLE 5:** Delivered acetone to the basolateral compartment (2 mL) of 6-transwell (MFC 2 LPM; 1-h exposure, 30 mL/min pump flow-rate) (n = 5).

Trans-well	1	2	3	4	5	6	Mean	SD	CV	Impinger conc	Total mass in impinger	Total air volume sampled	Air conc	ppm in air	Delivery
Exp	ppm	ppm	ppm	ppm	ppm	ppm	ppm	​	%	ppm	mg	m^3^	mg/m^3^	ppm	%
1	2,278	1,173	1,532	2078	1978	1808	1901	265	13.96	2,968	59.36	0.0018	32,978	13,883	13.7
2	2,403	2,328	2,178	1983	2,138	2,358	2,231	160	7.17	3,018	60.36	0.0018	33,533	14,117	15.8
3	2,918	2,673	3,068	2,178	2,773	2,327	2,656	343	12.92	4,158	83.16	0.0018	46,200	19,449	13.7
4	2,940	2,416	2,353	3,184	2,228	2040	2,527	441	17.44	5,134	102.68	0.0018	57,044	24,014	10.5
5	2,753	2,290	2,197	2,353	3,034	1853	2,413	320	17.39	4,021	80.42	0.0018	44,678	18,808	12.8
​	2,658 ±302	2,176 ±580	2,266 ±548	2,355 ±483	2,430 ±451	2077 ±258	2,345 ±293	​	13.78 ±4,2	3,859.8 ±900.3	77.2 ±18.0	0.0018	42,887 ±10,003	18,054 ±4,211	13.3 ±1.9

SD, standard deviation; CV, coefficient of variation; % Delivery = (mean concentration of tanswell (ppm)/(ppm in air) x 100%.

**TABLE 6:** Delivered acetone to the basolateral compartment (2 mL) of 6-transwell (MFC 0.5 LPM; 1-h exposure; 30 mL/min pump flow-rate) (n = 5).

Trans-well	1	2	3	4	5	6	Mean	SD	CV	Impinger conc	Total mass in impinger	Total air volume sampled	Air conc	ppm in air	Delivery
Exp	ppm	ppm	ppm	ppm	ppm	ppm	ppm	​	%	ppm	mg	m^3^	mg/m^3^	ppm	%
1	1,009	1,567	1,231	1,094	1,517	1,046	1,244	243	19.56	1,517	30.34	0.0018	16,856	7,096	17.5
2	1,307	1,269	1,182	787	1,144	1,022	1,119	191	17.06	​	​	0.0018	​	​	-
3	1,132	1,182	1,467	1,367	1,072	1,194	1,236	150	12.16	1982	39.64	0.0018	22,022	9,271	13.3
4	749	1,079	762	912	897	802	864	117	13.59	1812	36.24	0.0018	20,133	8,476	10.2
5	1,109	712	884	799	959	774	873	145	16.56	1887	37.74	0.0018	20,967	8,826	9.9
​	1,061 ±205	1,162 ±310	1,105 ±283	992 ±243	1,118 ±243	968 ±177	1,067 ±188	​	15.8 ±2.9	17,521 ±521	36.0 ±4.0	0.0018	1994 ±2,230	8,417 ±939	13.9 ±3.2

SD, standard deviation; CV, coefficient of variation; % Delivery = (mean concentration of tanswell (ppm)/(ppm in air) × 100%.

**TABLE 7:** Delivered acetone to the basolateral compartment (2 mL) of 6-transwell. (MFC 0.2 LPM, 1-h exposure, 30 mL/min pump flow-rate).

Trans-well	1	2	3	4	5	6	Mean	SD	CV	Impinger Conc	Total mass in impinger	Total air volume sampled	Air conc	ppm in air	Deposition
Exp	ppm	ppm	ppm	ppm	ppm	ppm	ppm	​	%	ppm	mg	m^3^	mg/m^3^	ppm	%
1	1,450	2,570	1,450	1,550	1,550	1950	1753	441	25.15	4,420	88.4	0.0018	49,111	20,674	8.5
2	2,440	2,440	2,840	2,740	2,740	1,550	2,508	262	14.49	6,900	138	0.0018	76,667	32,274	7.8
3	2050	2,740	3,130	2050	2,340	1850	2,360	488	20.67	7,590	151.8	0.0018	84,333	35,502	6.6
4	1,692	2092	2,392	2092	2,192	2,882	2,224	395	13.59	6,200	124	0.0018	68,889	29,000	7.7
5	2,592	2,682	2,392	2,392	2,392	2,782	2,539	172	6.76	7,042	140.84	0.0018	78,244	32,939	7.7
​	2045 ±483	2,505± 258	2,441 ±636	2,165 ±441	2,243 ±436	2,203 ±594	2,277 ±319	​	16.1 ±7.1	6,430.4 ±1,228	129 ±24.6	0.0018	71,448 ±13,647	30,078 ±5,745	7.7 ±0.7

SD, standard deviation; CV, coefficient of variation; % Delivery = (mean concentration of tanswell (ppm)/(ppm in air) x 100%.

### 3.4 Delivery of benzyl alcohol to 6-transwell

When we tested benzyl alcohol delivery to the basolateral compartment of ALI at flow rates of 30 mL/min for 2 h and 1 h, the delivered amounts were 61.1 ± 24.1 and 35.5 ± 21.7 ppm, respectively, which were lower than those of isopropyl alcohol and acetone. The CV for inter-transwell delivery was 18.8% and 15.56%, respectively (Tables 8, 9). The percentages of delivery were 476% ± 529% and 691% ± 1,221% for 2 h and 1 h, respectively. Because of its high viscosity, semi-VOC nature, and strong tendency to adsorb onto walls and tubes, benzyl alcohol air concentrations could not be readily estimated from impinger concentrations.

**TABLE 8:** Delivered benzyl alcohol to the basolateral compartment (2 mL) of 6-transwell. SD, standard deviation; CV, coefficient of variation (3 LPM MFC, 2 h exposure, 30 mL/min flow-rate).

Trans-well no.	1	2	3	4	5	6	Mean	SD	CV	Impinger conc	Total mass in impinger	Total air volume sampled	Air conc	ppm in air	Delivery
Exp	ppm	ppm	ppm	ppm	ppm	ppm	ppm	​	%	ppm	mg	m^3^	mg/m^3^	ppm	%
1	81	82	78	88	104	88	86.8	9.3	10.71	58	1.16	0/0018	644/44	145.7	59.6
2	86	83	75	107	87	81	86.5	10.9	12.62	80	1,6	0/0018	888.89	201.0	43.0
3	83	67	73	68	51	68	68.3	10.39	15.18	9.1	0.18	0/0018	0.0036	22.9	298
4	45	52	58	40	47	54	49.3	6.56	13.30	1.5	0.03	0/0018	16.67	3.77	1,307
5	25	24	25	18	20	40	25.3	7.74	30.54	1,5	0.03	0/0018	16.67	3.77	670
6	47	54	51	52	40	38	47	6.62	14.1	20.8	0.42	0/0018	231.1	52.25	90.-
Mean±	61.2 ±25.5	57.8 ±23.8	58.2 ± 21.6	59.7 ±33.9	62.7 ±30.1	67.2 ±17.7	61.1 ±24.1	​	16.1 ±7.2	33.9 ±33.7	0.364 ±0.47	0.0018	231 ±360	71.5 ±82.7	476 ±529

SD, standard deviation; CV, coefficient of variation; % Delivery = (mean concentration of tanswell (ppm)/(ppm in air) x 100%.

**TABLE 9:** Delivered benzyl alcohol to the basolateral compartment (2 mL) of 6-transwell. (3 LPM MFC, 1 h exposure, 30 mL/min flow-rate).

Trans-well	1	2	3	4	5	6	Mean	SD	CV	Impinger conc	Total mass in impinger	Total air volume sampled	Air conc	ppm in air	Delivery
Exp	ppm	ppm	ppm	ppm	ppm	ppm	ppm	​	%	ppm	mg	m^3^	mg/m^3^	ppm	%
1	27	22	19	20	19	27	22.33	3.78	16.91	0.2	0.004	0/0018	2.22	0.50	4,466
2	17	20	24	18	26	22	21.17	3.49	16.48	1.2	0.024	0/0018	13.33	3.01	703
3	20	24	23	18	19	22	21.0	2.37	11.27	0	0	0/0018	0	0	-
4	9	18	19	20	21	24	18.5	5.09	27.51	4.2	0.084	0/0018	46.67	10.55	175
5	9	18	19	19	20	23	18.0	4.73	26.29	1.2	0.0024	0/0018	13.33	3.01	598
6	25	19	24	22	29	26	22.5	3.02	13.41	0	0	0/0018	0	0	-
7	21	14	14	17	11	19	16.0	3.69	23.05	0	0	0/0018	0	0	-
8	28	34	26	30	19	35	28.67	5.85	20.42	5.2	0.014	0/0018	57.78	13.06	220
9	27	34	20	29	28	23	26.83	4.88	18.17	2.2	0.044	0/0018	24.44	5.53	485
10	46	47	51	38	45	36	43.83	5.71	13.02	2.2	0.014	0/0018	24.44	5.53	793
11	33	33	38	40	33	34	35.17	3.06	8.70	20.2	0.404	0/0018	224.44	50.74	69.3
12	43	34	36	30	32	30	34.17	4.92	14.39	2.2	0.044	0/0018	24.44	5.53	618
13	62	72	68	65	74	70	68.50	4.46	6.51	53.2	1.064	0/0018	591.1	133.65	51.2
14	79	80	76	81	77	85	79.67	3.20	4.02	56.2	1.124	0/0018	624.44	141.18	56.4
15	69	68	72	89	88	68	75.67	10.05	13.29	53.2	1.064	0/0018	591.11	133.74	59.6
​	34.3 ±21.3	35.8 21.4	35.3 ±21.2	35.7 ±23.6	36.1 ±24.1	36.3 ±20.6	35.5 ±21.7	​	15.56 ±6.8	13.4 ±21.7	0.26 ±0.44	0.0018	149 ±241	33.7 ±54.5	691 ±1,221

SD, standard deviation; CV, coefficient of variation; % Delivery = (mean concentration of tanswell (ppm)/(ppm in air) x 100%.

### 3.5 Overall evaluation of delivery to 6-transwell

**FIGURE 6:**
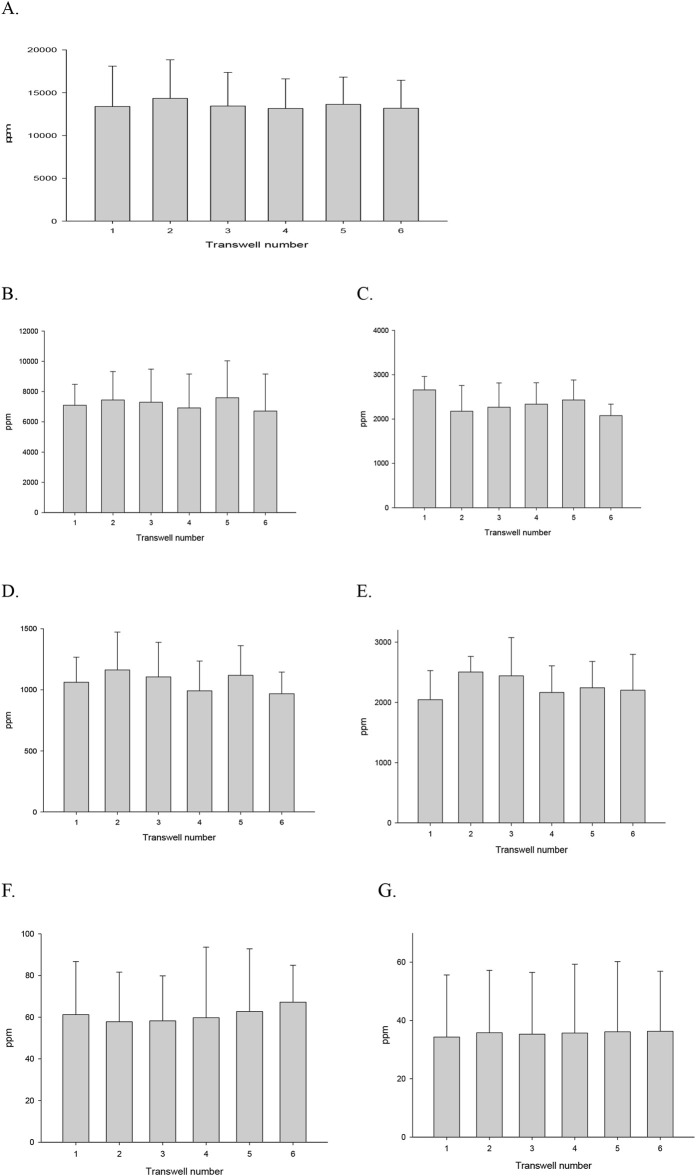
Deposition of IPA, Acetone, and Benzyl alcohol to ALI of 6-transwell. **(A)** IPA (3 LPM, 2 h exposure). **(B)** Acetone (3 LPM, 1 h exposure). **(C)** Acetone (2 LPM, 1 h exposure). **(D)** Acetone (0.5 LPM, 1-h exposure). **(E)** Aceton (0.2 LPM, 1-h exposure). **(F)** Benzyl alcohol (3 LPM, 2-h exposure). **(G)** Benzyl alcohol (3 LPM, 1-h exposure).

Figure 6 shows the overall inter-transwell VOC delivery in the 6-transwell. The coefficient of variation (CV) quantifies the relative variability of a dataset around its mean. Also, it indicates how consistent or uniform your measurements are, independent of their absolute magnitude. As shown in Table 10, ANOVA revealed no statistically significant differences among the six transwells, indicating consistency across flow rates from 2 LPM to 0.2 LPM, regardless of the test VOCs. It showed statistically uniform delivery. CV values ranged from approximately 7%–19% for most exposure conditions, consistent with reported variability in ALI exposure systems, and did not result in statistically significant positional differences. Our CV ranging 9.96%–18.8% indicates that the transwell system distributes delivery evenly, supporting its use in VOCs like isopropyl alcohol and acetone, and semi-volatile VOCs such as benzyl alcohol exposure studies without concern for positional bias.

**TABLE 10:** Summary table for VOC deposition.

Table	Compound	Flow rate (MFC) LPM	Exposure duration	Pump flow (mL/Min)	Experiment repeat	Overall mean ± SD (ppm)	CV (%)	F-statistic	p-value	Result
3	IPA	3	2 h	30	12	13,491 ± 3,712	9.96	0.161	0.976	No significant difference
4	Acetone	3	1 h	30	9	7,196 ± 1808	11.73	0.207	0.958	No significant difference
5	Acetone	2	1 h	30	5	2,345 ± 293	13.78	1.02	0.426	No significant difference
6	Acetone	0.5	1 h	30	5	1,067 ± 188	15.8	0.467	0.797	No significant difference
7	Acetone	0.2	1 h	30	5	2,277 ± 319	16.1	0.629	0.679	No significant difference
8	Benzyl alcohol	3	2 h	30	6	61.1 ± 24.1	18.8	0.108	0.990	No significant difference
9	Benzyl alcohol	3	1 h	30	15	35.5 ± 21.7	15.56	0.015	≈1.000	No significant difference

## 4 Discussion

### 4.1 Inter-transwell delivery variation in vitro inhalation studies

A uniform and reproducible delivered concentration is a fundamental requirement for air–liquid interface (ALI) *in vitro* inhalation models, particularly when such models are used within new approach methodology (NAM)-based inhalation testing frameworks. While ALI platforms have been extensively validated for particulate and nanoparticle aerosols, comparatively little is known about the delivery behavior and spatial uniformity of volatile organic compounds, which exhibit rapid evaporation and diffusion-dominated transport. The present study addresses this gap by systematically quantifying inter-transwell variability in delivery for representative volatile and semi-volatile solvents under controlled exposure conditions.

The results of ten *in vivo* lung deposition studies conducted with previously silver nanoparticles ranged from 11 to 20 nm, gold nanoparticles 11–105 nm, and multiwalled carbon nanotubes 1,015 nm exposed to 4-6 rats for 6-h in a nose-only inhalation chamber resulted in mean CVs 12.31%, ranging from 6.1% to 21.7% (Lee et al., 2025a). The average percentage of studies evaluating particle-based ALI exposure systems has reported CVs of approximately 10%–25% across transwells under controlled conditions. Lee et al. (2025a) studied an *in vitro* ALI deposition study using NaCl nanoparticles ranging from 62 to 66 nm, resulted 6.25% and 6.95% CV, with 30 mL/min and 12 mL/min pump flow-rate, respectively. Lee et al. (2025b) also tested ALI deposition of median-size 128 nm protein nanoparticles for inter-transwell deposition. Their study reported CVs of 19.59% and 19.07% of CV at pump flow rates of 12 mL/min and 30 mL/min, respectively. The study by Lenz et al. (2009), which used a flow-through ALI exposure system for nanoparticles, reported well-to-well mass deposition variability of approximately 10%–15%, attributed primarily to small differences in local airflow and particle diffusion. Similarly, Aufderheide and Scheffler (2013) demonstrated that uniform aerosol distribution in multi-insert ALI systems is achievable but requires precise flow control and system calibration, with reported inter-insert variability typically remaining below 20%. Electrostatic precipitation-based ALI systems, such as those incorporating electrostatic enhancement to increase deposition efficiency, have also been evaluated for spatial uniformity. De Bruijne et al. (2009) showed that although electrostatic forces substantially increase the deposited dose, spatial heterogeneity can arise when electric field strength or grounding differs among inserts, leading to increased inter-well variability. Consequently, many studies emphasize the importance of independently validating dose delivery to each insert rather than relying on chamber-averaged concentrations.

Particle size and deposition mechanism strongly influence inter-transwell variability. Diffusion-dominated deposition, characteristic of ultrafine particles and vapors, generally produces more homogeneous distribution across inserts than impaction- or sedimentation-driven deposition of larger particles (Jeannet et al., 2015; Rostami, 2009; Lee et al., 2025b). Computational fluid dynamics (CFD) simulations coupled with experimental validation have demonstrated that even minor flow asymmetries can disproportionately affect the deposition of larger aerosols, whereas gas-phase diffusion tends to smooth spatial gradients (Rostami, 2009). When Lee et al. (2025b) evaluated several deposition mechanisms, diffusion, impaction, gravitational settling, electrostatic deposition, and thermophoretic deposition of 125 nm median size of protein particles to a 6-transwell system, the flow-rates used in that study (12–30 mL/min) exclude impaction and gravitational settling, but mostly diffusion-dominant deposition operated in those settings.

Despite these advances, standardized acceptance criteria for inter-transwell variability are not universally defined. However, several inhalation toxicology reviews and dosimetry workshops suggest that inter-tanswell CV values ≤ 10–20% are generally acceptable for *in vitro* ALI exposure systems, provided that no statistically significant positional bias is observed (Lenz et al., 2009; Kleinstreuer et al., 2016). Reporting inter-transwell variability alongside deposited dose metrics is increasingly recommended to enhance transparency and reproducibility across studies.

In this context, the variability in inter-transwell delivery to the basolateral compartment observed in the present study (CV approximately 7%–19%, depending on compound and exposure conditions) is comparable to or lower than values reported for many particle-based ALI systems. The absence of statistically significant differences across transwell positions indicates a stable airflow distribution and reproducible dose delivery. These findings support the suitability of diffusion-dominated ALI exposure approaches for volatile and semi-volatile organic compounds and align with current expectations for acellular *in vitro* inhalation exposure systems used to support NAM-based assessments.

### 4.2 Diffusion-driven delivery of volatile solvents at the ALI

This study demonstrates that volatile organic solvents with high vapor pressures, such as isopropyl alcohol (IPA) and acetone, can be reproducibly delivered onto an air–liquid interface (ALI) under controlled *in vitro* exposure conditions. Although these solvents rapidly evaporate following atomization, their delivery onto the aqueous ALI is governed primarily by gas-phase diffusion rather than by inertial impaction or gravitational settling. Highly water-miscible aerosols and vapors readily dissolve in the liquid phase at the ALI, consistent with classical gas–liquid mass-transfer theory and pulmonary-uptake models (Fiserova-Bergerova, 1983; Rostami, 2009).

The relatively low coefficients of variation (CV < 10% for IPA and <16% for acetone) indicate that diffusion-driven transport produces spatially uniform solvent delivery across the six transwells. This behavior contrasts with particulate aerosols, for which deposition is often dominated by aerodynamic size, electrostatic effects, and flow maldistribution, frequently resulting in higher inter-well variability (Lenz et al., 2009; Aufderheide and Scheffler, 2013).

### 4.3 Influence of physicochemical properties on delivery efficiency

Differences in the delivered concentrations of IPA, acetone, and benzyl alcohol are consistent with their vapor pressures, evaporation rates, and viscosities. Acetone, with a vapor pressure approximately four times higher than that of IPA, exhibited lower delivery efficiency and modestly increased variability, reflecting greater evaporative loss during aerosol generation and transport. Similar observations have been reported in inhalation studies of highly volatile solvents, in which rapid vapor-phase dilution reduces the effective dose at the epithelial surface (Fiserova-Bergerova and Hughes, 1969; Morris et al., 1996).

Benzyl alcohol, a semi-volatile organic compound with low vapor pressure and high viscosity, displayed lower delivery but maintained CV values below 20%. Elevated viscosity affects aerosol formation by promoting larger, less stable droplets and increasing wall losses in tubing and exposure chambers. High-viscosity organic aerosols also exhibit slower internal diffusion and altered gas–particle partitioning, which can limit evaporation and condensation dynamics (Shiraiwa et al., 2013). These mechanisms likely contributed to the lower and more variable benzyl alcohol delivery observed here, particularly at longer exposure durations.

### 4.4 Effect of generation flow rate on delivery uniformity

The nonlinear relationship between acetone delivery and generation flow rate suggests a balance between aerosol residence time, dilution, and evaporation. At higher generation flows, increased aerosol concentration improves delivered concentration, whereas at very low flows, prolonged residence time may enhance diffusive transport despite lower source strength. Similar tradeoffs have been described in ALI exposure systems for volatile and semi-volatile compounds, where both airflow and exposure duration critically determine delivered dose (de Bruijne et al., 2009; Jeannet et al., 2015). Importantly, across all tested flow rates (0.2–3 L/min), no statistically significant inter-transwell differences were observed, demonstrating that the HIVIS system provides stable, well-distributed airflow. This robustness is essential for ensuring reproducible dose delivery *in vitro* inhalation studies intended to support NAM-based inhalation testing.”

### 4.5 Implications for *in vitro* inhalation testing and NAM-supportive dosimetry

Uniform deposition or delivery across exposure wells is a key requirement for *in vitro* inhalation test systems used within new approach methodologies (NAMs), as spatial dose variability can confound concentration–response relationships and biological interpretation. The CV values observed in this study (approximately 7%–19%) are comparable to, or better than, those reported for many particle-based ALI systems and fall within acceptable experimental variability for *in vitro* inhalation testing (Lenz et al., 2009; Lucci et al., 2018).

By demonstrating consistent delivery of volatile and semi-volatile solvents without electrostatic enhancement or complex aerosol conditioning, this study supports the applicability of diffusion-dominated ALI exposure for organic vapors. Coupled with real-time PID monitoring and post-exposure chemical quantification, this approach enables quantitative dosimetry, a critical component supporting the regulatory acceptance of NAM-based *in vitro* inhalation test systems (Kleinstreuer et al., 2016; Lee et al., 2025a).

### 4.6 Limitations and future directions

This study employed an acellular ALI model to isolate physicochemical delivery behavior. The presence of cells, mucus layers, or surfactant may alter solvent uptake kinetics and retention. Additionally, temperature and relative humidity were not actively controlled, though both parameters strongly influence evaporation and gas–liquid partitioning of VOCs (Rostami, 2009). Also, our concentration levels were very high, not the human-exposed concentrations. The diffusion-driven VOC delivery should operate at both low and high concentration levels. Future work integrating controlled environmental conditions and computational fluid–dynamic modeling would further refine dose prediction and enhance in vitro–in vivo extrapolation. Accordingly, the present findings should be interpreted as providing exposure and dosimetry context rather than toxicological hazard characterization.

## Data Availability

The original contributions presented in the study are included in the article/supplementary material, further inquiries can be directed to the corresponding author.
